# Student Perspectives on the Undergraduate Medical Student Research Experience at a Single United States Allopathic Institution

**DOI:** 10.7759/cureus.60809

**Published:** 2024-05-21

**Authors:** Michael A Deleon, Simren Lakhotia, Jonathan Gelfond, Courtney Peebles, Kate Lathrop, Sylvia Botros-Brey

**Affiliations:** 1 Office of Research, University of Texas Health Science Center at San Antonio, San Antonio, USA; 2 Population Health Sciences, University of Texas Health Science Center at San Antonio, San Antonio, USA; 3 School of Public Health, University of Texas Health Science Center at San Antonio, San Antonio, USA; 4 Obstetrics and Gynecology, University of Texas Health Science Center at San Antonio, San Antonio, USA

**Keywords:** medical student research, survey research, medical school education, step 1 change, electronic residency applications

## Abstract

Introduction

The purpose of this study was to identify student-reported institutional facilitators and barriers to successful research experiences at a single United States allopathic institution. Residency applications have increasingly become more competitive, and with the United States Medical Licensing Examination (USMLE) Step 1 exam’s transition to pass/fail, factors such as research experience and outcomes may become more important to increase residency application competitiveness. This study sought to explore factors that impact successful research experiences leading to tangible outcomes for medical students at our medical school, the Joe R. & Teresa Lozano Long School of Medicine.

Methods

A cross-sectional survey was developed and administered via REDCap to 853 students in May 2022. Survey question domains included demographics, past and present research participation, perceived barriers/facilitators to research, tangible outcomes (e.g., publications and posters), and overall satisfaction with research comparing subjectively "best" and "worst" experiences. The Institutional Review Board (IRB) deemed this project as non-regulated research.

Results

We had a 24% (n = 204/853) response rate. The responses were distributed equally among the four classes. A big portion of the participants (71%, n = 59/83) identified a tangible outcome as the most important measure of success. Regarding facilitators, students identified having a mentor (89%, n = 165/184) and departmental connections (85%, n = 156/184) as the most important when looking for a project. Barriers included SMART goals (Specific, Measurable, Achievable, Relevant, and Time-Bound) lacking in 31% (n = 24/75) of worst projects, followed by a clear timeline in 29% (n = 22/76) and hours of commitment in 27% (n = 21/78). The best projects were more likely to have resulted in a publication (61% (27/44) vs. 32% (14/44)) or have a poster (64% (28/44) vs. 36% (16/44)).

Conclusions

Medical students are interested in participating in research, with important facilitators including mentorship and departmental connections. Modifiable variables include lack of clear timelines, well-defined roles and responsibilities, and time commitments. This information may be useful for faculty who mentor medical students or medical schools interested in designing medical student research programs.

## Introduction

In the United States, the selection process for residency interviews and positions has become more competitive over the last six years [1-3]. Arguably, one of the most important components of the residency application is the United States Medical Licensing Examination (USMLE) Step 1 exam, which has historically been a significant predictor of performance on future board exams. These exam scores are noted by residency programs across specialties as one of the main drivers for selecting applicants for interviews because the USMLE objectively compares students from different institutions across the country [4-8]. As of January 26, 2022, Step 1 has transitioned to a pass/fail scoring system. This change may dramatically impact the residency matching process by reducing the standardization of medical students’ applications, placing more emphasis on other components of the residency application [9-13].

The Electronic Residency Application Service (ERAS) is the primary method of applying to residency programs and encompasses standardized exam scores, letters of recommendation, and extracurriculars, among other factors. We hypothesize that other aspects of residency applications, particularly research experiences and achievements, may increase in importance for residency programs as a result of the changes in the assessment of applicants.

Participating in research during medical school has proven to be a critical component of an applicant’s competitiveness, particularly when applying to competitive specialties [14]. Based on the 2016 and 2018 US residency match outcomes, students who successfully matched to a residency had a significantly greater number of research experiences than those who did not match [14]. Since 2016, the mean number of research experiences has increased by 33%, and the mean number of abstracts/posters/publications has increased by 68% among matched applicants [1-3].

The sections of the ERAS application pertaining to research involvement include "research experiences" and "publications," which outline the credit received for an individual's work [15]. Under the "research experiences" section, students may choose to use their limited number of entries to describe their involvement/work in research projects. Under the "publications" section, students may list all tangible outcomes or deliverables. 

There is a paucity of research evaluating medical student research experiences. One study identified the barriers that students face when seeking research opportunities in an osteopathic institution to include lack of time and feeling overwhelmed and unsure about how to start and how to access research. This study found the lack of research infrastructure as a notable factor [16]. Our institution, the Joe R. and Teresa Lozano Long School of Medicine (LSOM), does have an established research infrastructure with access to research for students. The purpose of this study was to analyze student-reported facilitators and barriers to research by surveying medical students enrolled at our medical school.

## Materials and methods

Rationale

The first authors of this study work directly with the LSOM Office of Research as medical student research liaisons. This position's responsibilities are multifold, with the goal being to help facilitate medical student research by providing insight and feedback to the Office of Research. Serving in this role, paired with the new change to pass/fail, we began to discuss its potential implications with peers, faculty, researchers, and administrators from various departments at our institution. Based on these informal discussions, we hypothesized that participating in research may become more important.

Study design

We sought to understand our peers’ perspectives of facilitators and barriers to successful research experiences. This was done by conducting a cross-sectional survey, distributed to all four classes of medical students at our allopathic school of medicine. This study was determined by the University of Texas Health Science Center San Antonio Institutional Review Board to be non-regulated research because it is not regulated research as defined by DHHS regulations at 45 CFR 46 and FDA regulations at 21 CFR 56. Participating in and completing the survey implied consent.  

Survey design

We developed a REDCap online survey using a systematic process for designing high-quality questionnaires including reviewing and synthesizing the literature, developing items, collecting feedback through expert validation, employing cognitive interviews, and conducting pilot testing (see Appendix) [17-19]. Question types included multiple choice, Likert scales, rank orders, and free text. The survey began with an introductory page delineating the reason for conducting the research, how the data would be used, and assurances of anonymity. The content of the survey included questions pertaining to interest in participation in research, prior experience in research, and further details describing individual research experiences and tangible outcomes, including abstracts, posters, and publications. Students were asked to identify the “best” and “worst” project experiences and to describe their experience working on these projects. The survey contained questions pertaining to project completion/incompletion, the project’s outcome, and credit received. Additional questions regarding the logistical aspects of the project were included to identify potential discrepancies between the best and worst projects. These included overall enjoyment, implementation of a clear timeline and/or roles and responsibilities, and well-defined goals while working on the project. The survey concluded with a series of questions requesting demographic information.

The survey was reviewed and revised for content validity by medical education content experts. After the development of the survey, a convenience sample of eight students, two from each graduating class, were asked to take the survey from multiple perspectives. This allowed for cognitive pretesting to be conducted from differing interpretations of the readability and flow of the survey design. Constructive feedback was obtained to introduce changes that subsequently led to improvements of the branching logic, structure, and functionality of the survey.

Dissemination and participants

All medical students enrolled at LSOM were invited by the Office of Research to participate in the survey through official class email list-servs. The REDCap survey was open from May 13 to 27, 2022. After initial distribution on May 13th, students received a follow-up reminder email three days prior to survey close. The participants were incentivized by providing them with an option to be entered in a class-wide raffle upon completion of the survey. There were four separate drawings, each for a $100 Amazon gift card (one per class). While participants were required to enter their email addresses to be entered into the raffle, email addresses were not connected to their survey responses.

Data analysis

Responses to Likert scale questions were described using frequencies and percentages for each categorical variable. We assessed the characteristics used to describe the best and worst project experiences within students who worked on two or more projects to understand impactful facilitators and barriers. Facilitators were identified as project characteristics that most often received a response of 5, or “highly desirable,” on a Likert desirability scale question that examined the perceived desirability of certain qualities in an ideal project. Barriers were identified by examining the delta of positive characteristics, looking at the difference in incidence of “rarely/never” responses on a Likert scale between best and worst projects. The McNemar test was used to test the best versus worst projects' differential rates of completion, enjoyment, and tangible outcomes (abstract, publication, and presentation) of the projects. All testing was two-sided at a statistical level of 0.05. Data analysis and creation of figures were performed using RStudio statistical software (R Core Team, R: A language and environment for statistical computing, R Foundation for Statistical Computing, Vienna, Austria) and Microsoft Office (Microsoft Corporation, USA).

## Results

Of the 853 medical students enrolled at our institution, 204 participated in our survey for an overall response rate of 24%. Table 1 demonstrates the demographics of our participants. The median age of the participants was 24 (interquartile range (IQR), 23.0-26.0). A majority of the participants were female (n = 95, 54%) and White (n = 90, 51%).

**Table 1:** Demographics of the survey participants IQR = interquartile range

Characteristic	Overall responses	Number of responses	Median (IQR)
Age	148		24.0 (23.0, 26.0)
Gender	176		
Female		95 (54.0%)	
Male		76 (43.2%)	
Race	176		
Asian		76 (43.2%)	
American Indian		2 (1.1%)	
Black or African American		6 (3.4%)	
Native Hawaiian or Other Pacific Islander		0 (0%)	
Other Race		12 (6.8%)	
White		90 (51.1%)	

Of all the respondents, 43 had completed a single project and 79 completed both a best and worst project. We examined the best and worst project characteristics within subjects who worked on two or more projects. The McNemar test was used to test the best versus worst projects' differential rates of completion, enjoyment, and reporting outcomes (abstract, publication, and presentation). All testing was two-sided at a statistical level of 0.05. 

Analysis of the best and worst projects is represented in Table 2. Student enjoyment was higher in the best project, with 92% (n = 73/79) reporting they enjoyed the best projects and 67% (n = 53/79) enjoying their worst project (p < 0.001). Students were more likely to complete the best projects, with a 46% (n = 36/79) completion rate versus 20% (n = 16/79) in the worst projects (p ≤ 0.001). Of those with deliverable outcomes, the best projects were more likely to have resulted in a publication (61% (n = 27/44) vs. 32% (n = 14/44) (p = 0.01)) or have a poster 64% (n = 28/44) vs. 36% (n = 16/44) (p = 0.01)). Similarly, the best projects had higher rates of presentation (39% (n = 17/44) vs. 16% (n = 7/44) (p = 0.01)), co-authorship (61% (n = 27/44) vs. 34% (n = 15/44) (p = 0.01)), main authorship (46% (n = 20/44) vs. 18% (n = 8/44) (p = 0.006)), while having statistically similar rates of abstracts (41% (n = 18/44) vs. 25% (n = 11/44) (p = 0.10)). 

**Table 2:** A comparison of participants' responses to questions about their best and their worst project's characteristics

Question	Answer	Best (Number of responses)	Worst (Number of responses)	p-value^1^	One project
Who did you report to?					
	PI	56 (63.6%)	42 (52.2%)		23 (53.5%)
	Resident	29 (33.0%)	15 (19.0%)		4 (9.3%)
	Medical Student	28 (30.7%)	24 (30.4%)		14 (32.6%)
	Another faculty	25 (28.4%)	14 (17.7%)		7 (16.3%)
Did the project go to completion?					
	Yes	39 (45.5%)	16 (20.2%)	<0.001	8 (18.6%)
	No	1 (1.1%)	13 (16.5%)		4 (9.3%)
	In progress	47 (52.3%)	39 (49.4%)		29 (67.4%)
Outcome					
	Publication	27 (61.4%)	23 (31.8%)	0.01	1 (12.5%)
	Poster	28 (63.6%)	32 (36.4%)	0.01	5 (62.5%)
	Abstract	18 (40.9%)	24 (25.0%)	0.10	4 (50%)
What credit did you receive?					
	Main author	20 (45.5%)	17 (18.2%)	0.006	0 (0%)
	Co-author	27 (61.4%)	35 (34.0%)	0.01	8 (100%)
	Presentation	17 (38.6%)	13 (15.9%)	0.01	0 (0%)
Did you enjoy the project?					
	Yes	83 (92.4%)	53 (67.0%)	<0.001	35 (81.4%)
	No	6 (7.6%)	26 (33.0%)		8 (18.6%)
1. McNemar’s Test					

**Figure 1:**
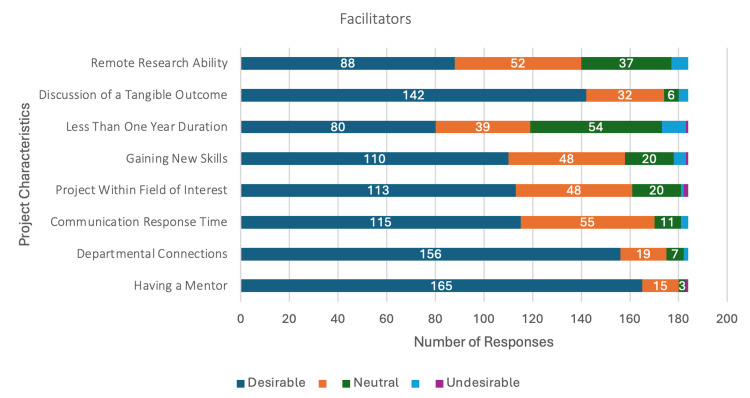
Distribution of responses to Likert scale questions about facilitating factors

Facilitators (Figure 1), such as having a mentor (n = 165/185, 89%) and departmental connections (n = 156/185, 85%), received the highest number of highly desirable responses. Discussion of a tangible outcome (n = 143/185, 77%) received the next highest number of highly desirable responses. The option to conduct research remotely was also identified as desirable in a research project (n = 89/185, 48%).

**Figure 2:**
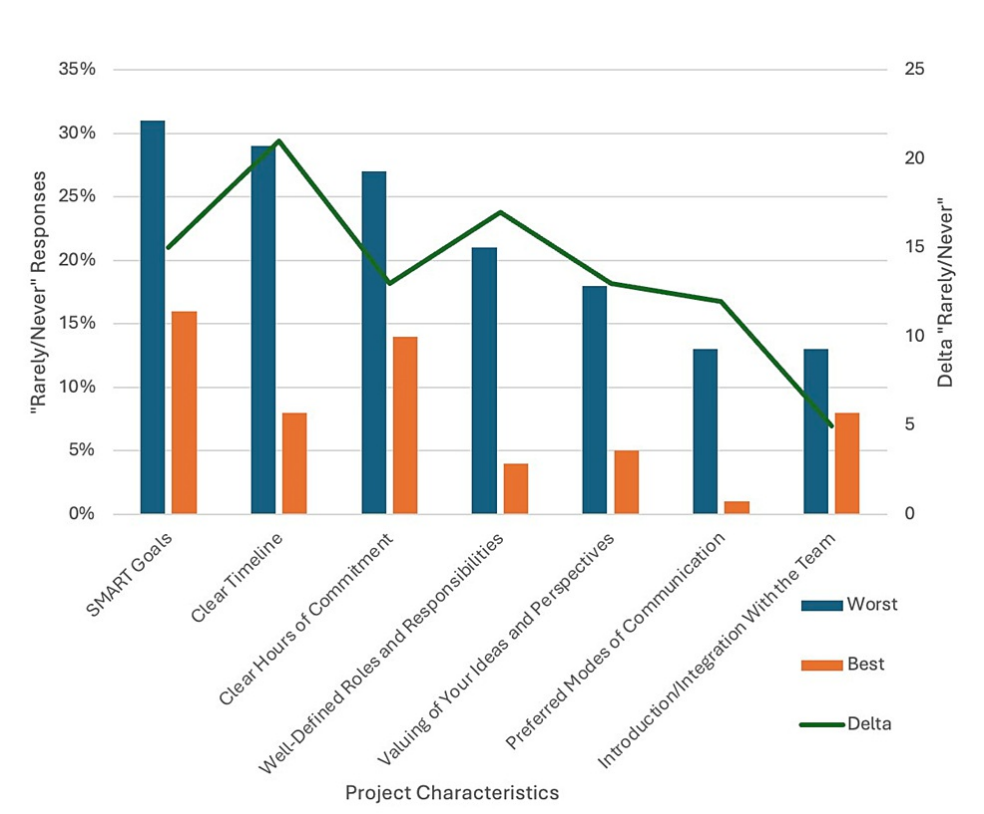
Frequency of "rarely/never" Likert scale responses to barrier factors Delta = number of "rarely/never" responses in the worst project; number of "rarely/never" responses in the best project

Barriers (Figure 2), such as SMART goals (Specific, Measurable, Achievable, Relevant, and Time-Bound), were lacking in 31% (n = 24/77) of the worst projects, followed by a clear timeline in 29% (n = 22/77) and hours of commitment in 27% (n = 21/77). When comparing these barriers across the best and worst projects, a clear timeline demonstrated the greatest difference in occurrence (Δ= 21). Well-defined roles and responsibilities had the next highest difference in occurrence between the best and worst projects (Δ = 17).

## Discussion

We found that medical students’ interest in participating in research at our institution is high with the most important facilitators being research mentorship, departmental connections, and discussion of tangible goals. Conversely, we identified that lack of clear timelines, well-defined roles and responsibilities, and clear time commitments were barriers to successful research experiences and tangible outcomes. Of these, the lack of a clear timeline was the most predictive of students having a poor research experience. These findings underscore the importance of effective project management, including faculty development and medical student education, in optimizing the research experience for medical students.

While there may be multiple factors that play into students’ success with a research experience, we found that students determine successful projects to be those that end in a tangible result. We suspect that this is likely because these tangible outcomes can be recorded in the ERAS application, strengthening the application. As the importance of research grows, it is now more important to understand ways to promote students’ success with research during their undergraduate medical education.

Much of the existing literature examining perceptions and attitudes toward research among medical students was completed before Step 1's transition to pass/fail or was limited to medical students outside of the United States [20-22]. Articles that did explore these factors within the US focused on one specialty and did not identify specific factors contributing to students’ success in completing research [23]. Ho et. al. identified facilitators and motivational factors that promote successful research endeavors within an osteopathic medical school with a limited research infrastructure [16]. One recent study surveyed students from a single community-based medical school regarding their perceptions toward research in light of the Step 1 scoring change, but this study did not seek to identify factors that facilitate and/or hinder their ability to complete research [11]. Furthermore, the authors of this article did not specify if their medical school was an allopathic or osteopathic institution. Our study further clarifies facilitators and barriers to medical student research and describes students' attitudes toward completing research within a US allopathic institution that provides research infrastructure. 

The results of this survey were discussed with institutional leadership, which supported the transition from a project-centered database to the initiation of developing a new student-mentor matching program. Students participate in this program by entering their names into a form, after which the Office of Research matches each student with a mentor looking to work with medical students. The mentorship program is meant to highlight students’ desire to find meaningful mentors and research projects that provide them with tangible outcomes.

The strengths of this study include its timeliness and relevance, which address students' perceptions of the transition to a pass/fail scoring system for the USMLE Step 1 exam and its potential implications for residency applications. The survey was validated with expert reviews, cognitive testing, and pilot testing. The findings of this study contribute to a gap in the literature regarding student perceptions and facilitators/barriers to student research. Limitations of this study include a low response rate leading to a response bias that may artificially increase the percentage of students interested in and participating in research. Our low response rate (24%) may have been impacted by the fact that our survey was distributed after match day, which could have led to decreased motivation to report feedback by fourth-year medical students. Furthermore, those who did respond do not reflect the diverse nature of our medical school class demographics. Clerkship duties, Step 1 exam preparations, and/or other medical school curricula may have also impacted students’ ability to complete the survey in a timely manner. In addition, reliance on self-reported data exposes the study to recall bias and social desirability bias, potentially affecting the accuracy of the results. The findings are specific to LSOM, limiting their generalizability to other medical institutions with different structures, resources, and cultures.

## Conclusions

The transition to a pass/fail scoring system for the USMLE Step 1 exam has prompted a reevaluation of the factors contributing to the competitiveness of medical residency applications. Our study emphasizes the increasing importance of studying the factors that impact medical students' ability to complete research. The identification of modifiable facilitators and barriers within our institution provides valuable insights for both medical students and institutions seeking to enhance the research engagement of their students. As the medical education landscape continues to evolve, including increased student interest in research, ongoing research and adaptability in supporting students' research endeavors will be essential.

## Figures and Tables

**Figure 3:**
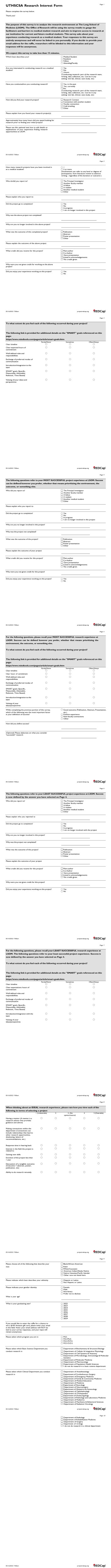
Sample survey form
